# Gallbladder volvulus with preoperative and intraoperative imaging

**DOI:** 10.1093/jscr/rjad048

**Published:** 2023-02-17

**Authors:** Andrew Stafford Beatty, Krish Kulendran, Harish Iswariah, Manju D Chandrasegaram

**Affiliations:** Department of General Surgery, The Prince Charles Hospital, Brisbane, Queensland, Australia; Northside Clinical School, School of Medicine, The University of Queensland, Brisbane, Queensland, Australia; Department of General Surgery, The Prince Charles Hospital, Brisbane, Queensland, Australia; Northside Clinical School, School of Medicine, The University of Queensland, Brisbane, Queensland, Australia; Department of General Surgery, The Prince Charles Hospital, Brisbane, Queensland, Australia; Northside Clinical School, School of Medicine, The University of Queensland, Brisbane, Queensland, Australia; Department of General Surgery, The Prince Charles Hospital, Brisbane, Queensland, Australia; Northside Clinical School, School of Medicine, The University of Queensland, Brisbane, Queensland, Australia

## Abstract

Volvulus of the gallbladder is one of the rarest conditions to affect the gallbladder, however, it should remain an important differential. Typically, it is diagnosed in elderly women, but it has also been reported in children and men. The lack of unique distinguishing features make diagnosis difficult to distinguish between other gallbladder pathology such as acute cholecystitis; however, delayed recognition or non-operative management is associated with higher mortality. We present the case of a 92-year-old woman who presented with this pathology, had diagnosis established preoperatively and was successfully treated with a cholecystectomy.

## INTRODUCTION

Like the sigmoid colon or stomach, the gallbladder has the potential to twist or volve. Gallbladder volvulus was first being described by Wendel in 1898 [1], and since then, >500 have been cases reported in the literature [2]. While there has been an increase in number of reported cases over recent decades, in part due to the association between the condition and advancing age, gallbladder volvulus only accounts for 1 out of 365 520 of all gallbladder-related pathologies presenting to hospital; despite this, it is an important differential that should not be overlooked [3]. Lack of distinguishing features from other biliary pathologies makes the diagnosis difficult, but lack of prompt recognition leads to an increased mortality risk that can be avoided with early surgical intervention [4]. We present a 92-year-old woman who was successfully diagnosed preoperatively and was appropriately treated by laparoscopic cholecystectomy.

## CASE REPORT

A 92-year-old woman presented to our hospital following a syncopal episode at home. She was diagnosed with new rapid atrial fibrillation with rate between 150 and 180 bpm. She had a past medical history of hypertension, ischaemic heart disease and Stage 4 chronic kidney disease. Bloods showed normal inflammatory markers and liver function tests but elevated troponins. She was diagnosed with a Type 2 myocardial infarction and was admitted under the cardiology team for rate control and further investigation.

**Figure 1:**
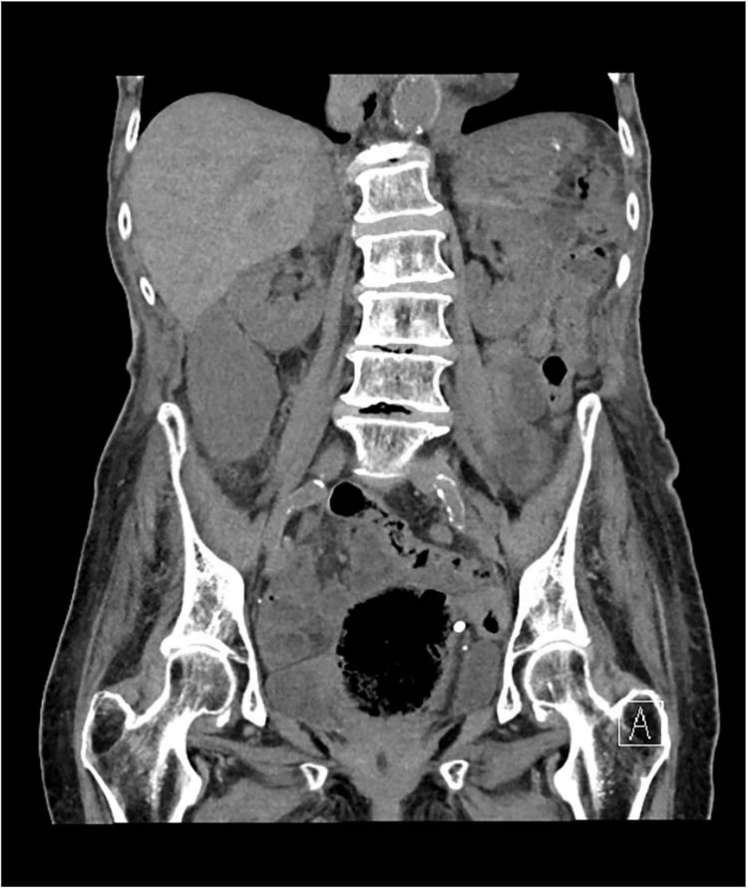
Axial slice of non-contrast CT abdomen and pelvis, demonstrating a distended gallbladder located outside the typical anatomical location, inferior the liver edge, causing medial displacement of the right kidney.

The morning after her admission, she began to complain of right-sided abdominal pain that coincided with a rise in her inflammatory markers. She proceeded for a non-contrast computed tomography (CT) of her abdomen and pelvis due to her poor renal function. The CT scan demonstrated a very distended gallbladder causing medial displacement of the right kidney and hepatic flexure of the ascending colon (Fig. 1). The gallbladder was inferior to the right liver lobe rather than the typical anatomical position within the gallbladder fossa and was suspicious for gallbladder volvuli.

**Figure 2:**
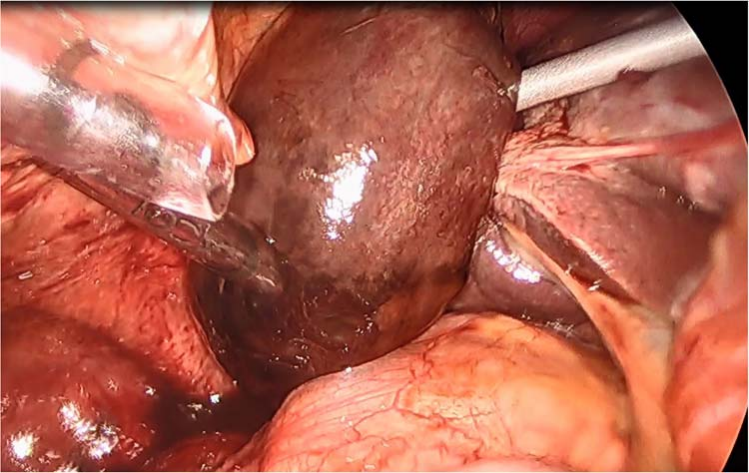
Intraoperative picture of a gangrenous, floating gallbladder.

**Figure 3:**
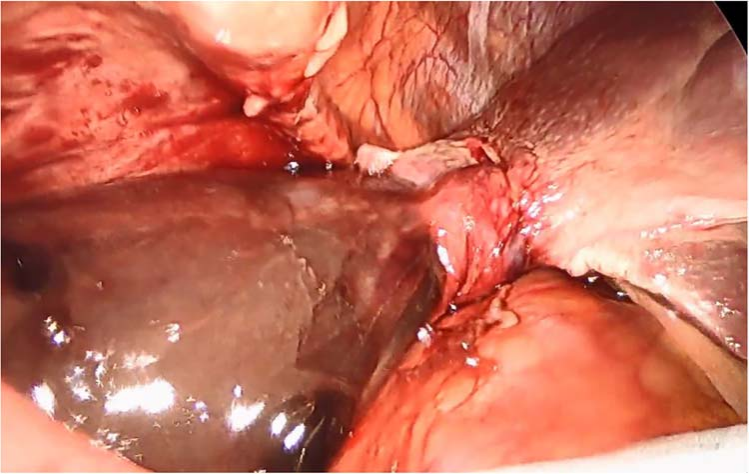
Intraoperative picture of the gangrenous gallbladder narrowing upon the torted pedicle; the cystic duct and artery.

**Figure 4:**
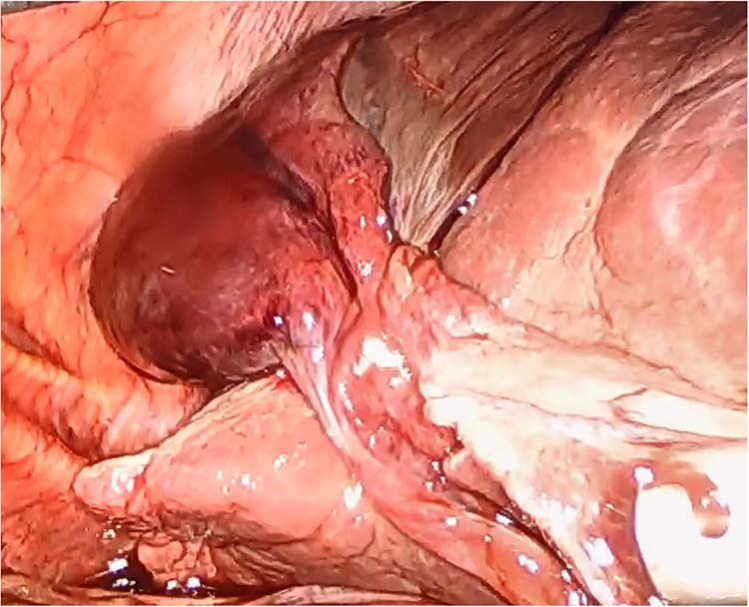
Gallbladder after being un-torted, showing the long cystic pedicle.

**Figure 5:**
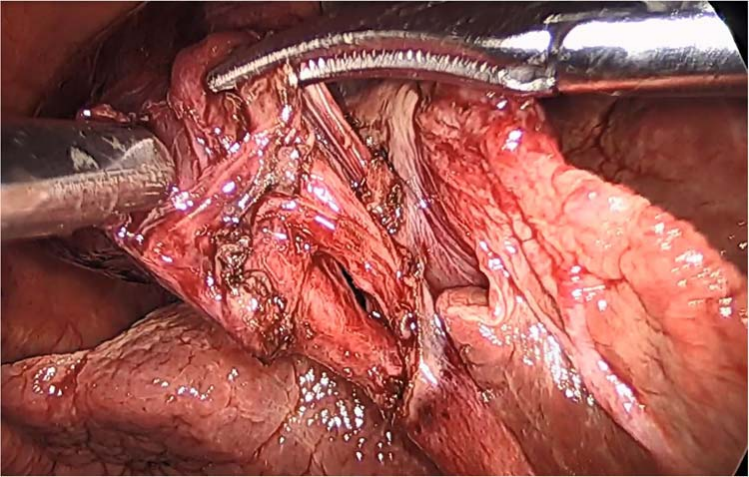
Intraoperative picture of the critical view following dissection of the cystic duct and artery.

**Figure 6:**
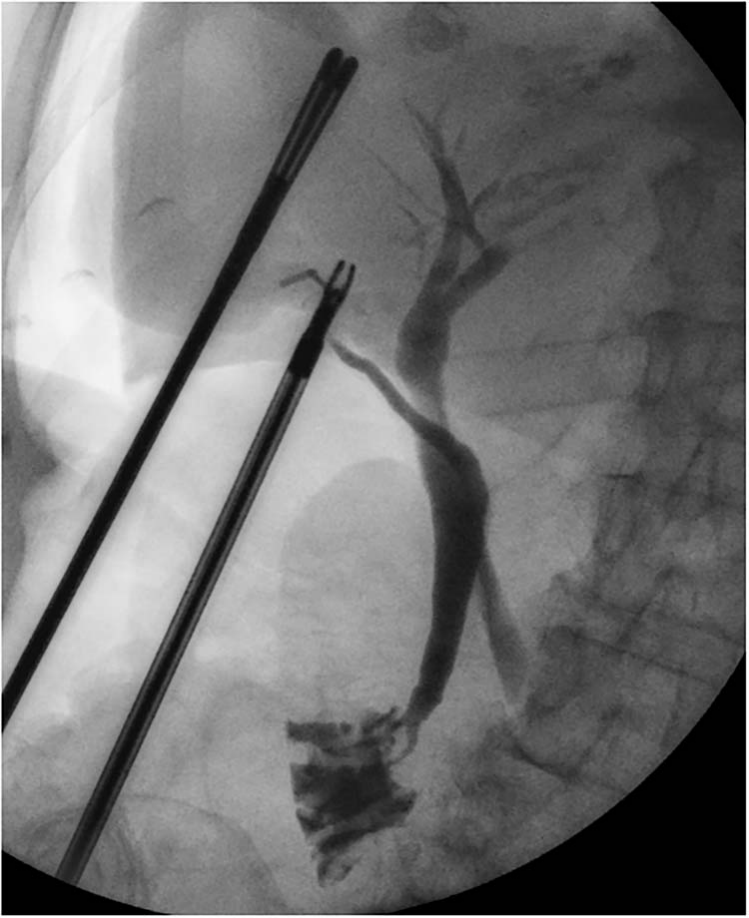
Intraoperative cholangiogram showing a long tortuous cystic duct with a medial insertion into the common bile duct.

She had a good functional baseline, living in home independently and following anaesthetic review; after a family discussion, the consensus was to proceed to surgery. She underwent a laparoscopic cholecystectomy, and intraoperatively, the diagnosis of gallbladder volvulus was confirmed. A distended gangrenous gallbladder was found, which had undergone 360° clockwise rotation about the cystic duct (Figs 2, 3). The gallbladder was detorted to facilitate a traditional dissection of Calot’s triangle, achieving the critical view of safety (Figs 4, 5). The cystic duct was able to be cannulated facilitating an intraoperative cholangiogram which was unremarkable (Fig. 6). The gallbladder was then removed, and operation was completed without any complications. The histopathology of the gallbladder found diffuse haemorrhagic necrosis of the gallbladder without any evidence of dysplasia or malignancy. No cholelithiasis was present.

Her recovery was complicated by a left parietal infarct, which was medically managed. Following an extended inpatient stay for rehabilitation, she was successfully discharged to an assisted living complex and there are no further issues.

## DISCUSSION

While gallbladder volvulus can occur at any age, there are two peak incidences: those <18 years of age and those >70 years of age, with the latter being far more predominant. Within the elderly population, prevalence is higher in women than men with a 5:1 ratio [3]. The presentation mimics that of cholecystitis and there are almost no distinguishing clinical features between the two pathologies.

Diagnosis has only been made preoperatively in 17% of cases [5], and while imaging has been essential for this, there are no data to compare modalities. Ultrasound doppler can assess the interrupted flow from the cystic pedicle [6]. Cross-sectional imaging has been proposed for the better assessment of the cystic duct, with magnetic resonance cholangiopancreatography being favoured over CT. Signs that have been reported include tapering and twisting of the cystic duct, differing uptake intensities of the cystic duct and gallbladder compared to extra hepatic ducts and V-shaped distortion of the extrahepatic bile duct due to traction [7]. While these signs can be evident, the most common findings are gallbladder distention, wall thickening and pericholecystic fluid, which are not specific for torsion [8].

A conservative approach with antibiotics is insufficient to treat a gallbladder volvulus, with eventual progression from ischaemia to necrosis and eventual perforation. If left untreated, the mortality is as high as 6% [5]. Delay of >2 days from the onset of symptoms to surgical intervention was associated with higher mortality [4]. Percutaneous cholecystostomy is discouraged due to the risk of biliary peritonitis either due to the lack of attachments to the liver surface or due to the eventual necrosis of the wall [9].

The development of gallbladder volvulus is likely multifactorial as opposed to a single unifying cause. Lack of attachments between gallbladder and cystic plate, elongated or tortuous cystic pedicle and lack of visceral fat are the commonly proposed contributing factors [10]. The presence of cholelithiasis is thought not to play a role in the development of this condition, given, in >75% of individuals, cholelithiasis is absent [9]. The torsion can be defined as complete or incomplete depending on the degree of rotation, be it > or <180°. It can also be classified according to the direction of rotation, with clockwise rotation being attributed to gastric hyperperistalsis and the anti-clockwise rotation being attributed to colonic hyperperistalsis [11]. Regardless of the rotational direction, the management is unchanged.

## CONCLUSION

Gallbladder volvulus can be an elusive diagnosis to make preoperatively. It requires a heighted index of suspicion, especially in elderly female patients presenting with acalculous cholecystitis. While imaging can be useful in establishing diagnosis, even cross-sectional imaging has its limitations. Failure to progress should raise clinical concern in all patients, with the acceptance that non-operative management is unlikely to prevail and early intervention by means of cholecystectomy is the only way to prevent the associated mortality.

## CONFLICT OF INTEREST STATEMENT

None declared.

## FUNDING

No funding was received for this case report.

## AUTHORS’ CONTRIBUTIONS

A.S.B. was responsible for the study concept, design and writing (original draft); K.K., H.I. and M.D.C. were in charge of review and editing; H.I. took care of the supervision.
